# Use of the Internet as a Health Information Resource Among French Young Adults: Results From a Nationally Representative Survey

**DOI:** 10.2196/jmir.2934

**Published:** 2014-05-13

**Authors:** François Beck, Jean-Baptiste Richard, Viet Nguyen-Thanh, Ilaria Montagni, Isabelle Parizot, Emilie Renahy

**Affiliations:** ^1^Institut National de Prévention et d’Education pour la Santé (INPES)ParisFrance; ^2^Cermes3 - Equipe Cesames (Centre de recherche Médecine, Sciences, Santé, Santé mentale, Société)Université Paris Descartes, Sorbonne Paris Cité /CNRS UMR 8211/Inserm U988/EHESS45, rue des Saints-Pères. 75270 Paris Cedex 06ParisFrance; ^3^Section of PsychiatryDepartment of Public Health and Community MedicineUniversity of VeronaVeronaItaly; ^4^Research Team on the Social Determinants of Health and HealthcareUMRS 707INSERMParisFrance; ^5^CNRS, Centre Maurice HalbwachsERISParisFrance; ^6^Centre for Research on Inner City HealthLi Ka Shing Knowledge InstituteSt. Michael's HospitalToronto, ONCanada

**Keywords:** health communication, information dissemination, access to information, Internet, trust, young adults

## Abstract

**Background:**

The Internet is one of the main resources of health information especially for young adults, but website content is not always trustworthy or validated. Little is known about this specific population and the importance of online health searches for use and impact. It is fundamental to assess behaviors and attitudes of young people looking for online health-related information and their level of trust in such information.

**Objective:**

The objective is to describe the characteristics of Internet users aged 15-30 years who use the Web as a health information resource and their trust in it, and to define the context and the effect of such use on French young adults’ behavior in relation to their medical consultations.

**Methods:**

We used the French Health Barometer 2010, a nationally representative survey of 27,653 individuals that investigates population health behaviors and concerns. Multivariate logistic regressions were performed using a subsample of 1052 young adults aged 15-30 years to estimate associations between demographics, socioeconomic, and health status and (1) the use of the Internet to search for health information, and (2) its impact on health behaviors and the physician-patient relationship.

**Results:**

In 2010, 48.5% (474/977) of Web users aged 15-30 years used the Internet for health purposes. Those who did not use the Internet for health purposes reported being informed enough by other sources (75.0%, 377/503), stated they preferred seeing a doctor (74.1%, 373/503) or did not trust the information on the Internet (67.2%, 338/503). However, approximately 80% (371/474) of young online health seekers considered the information found online reliable. Women (*P*<.001) and people with higher sociocultural positions (OR 0.5, 95% CI 0.3-0.9 and OR 0.4, 95% CI 0.2-0.7 for employees and manual workers, respectively, vs individuals with executive or manager positions) were more likely to use the Internet for health purposes. For a subsample of women only, online health seeking was more likely among those having a child (OR 1.8, 95% CI 1.1-2.7) and experiencing psychological distress (OR 2.0, 95% CI 1.0-4.0). Finally, for online health seekers aged 15-30 years, one-third (33.3%, 157/474) reported they changed their health behaviors (eg, frequency of medical consultations, way of taking care of one’s own health) because of their online searches. Different factors were associated with different outcomes of change, but psychological distress, poor quality of life, and low income were the most common.

**Conclusions:**

The Internet is a useful tool to spread health information and prevention campaigns, especially to target young adults. Young adults trust online information and consider the Internet as a valid source of health advice. Health agencies should ensure the improvement of online health information quality and the creation of health-related websites and programs dedicated to young adults.

## Introduction

### Background

The use of the Internet to look for advice or health information has been a growing resource since the 1990s [1]. Health prevention programs can benefit from the Internet especially when dedicated to or designed for young adults who represent the vast majority of Web users [2]. High-quality health information can be provided through websites, forums, blogs, and social networks, which have been some of the most popular channels for health promotion among young people in the past 10 years [2,3]. In France, 3 of 4 people have access to the Internet, and Internet use is higher in young people compared to other adults: 99% of people aged 12-17 years use the Internet, and this proportion falls to 22% for those aged 70 years and older [4]. Some French websites exclusively address health issues concerning either the general population or adolescents in particular. However, given the pace with which informal websites and blogs are created [5], young Web users do not exclusively use official websites whose content is trustworthy and certified by experts and quality labels [6,7]. For this reason, and given the amount of health information available on the Internet, it is fundamental to investigate behaviors and attitudes of young adults searching for health-related information on the Web (young online health seekers or aged 15-30 years health seekers). Therefore, we deemed it important to describe the profile of young online health seekers together with the context and consequences of their searches.

The Web offers a large amount of health-related information and benefits from different interactive formats. However, the disparate quality of available information [8-11] might reinforce social disparities among Web users [12]. This heterogeneity is also linked to the perception of reliability and credibility Web users have regarding the information found on the Internet [13]. The French National Authority for Health (Haute Autorité de Santé; HAS) pilots the certification procedure of health-related websites by using the Health On the Net (HON) Code [6]. However, this initiative does not provide a complete evaluation of all available information. According to the Pew Research Center’s Internet & American Life Project conducted in the United States in 2009, 3% of online health seekers stated they had health problems after having followed medical advice or information found on the Web [14]. Ambiguity regarding the quality of health information on the Web affects and worries some adult Web users [15]. Therefore, it is essential to understand what young people think about the credibility of online health information.

Furthermore, the effect of these searches on the way young people take care of their own health and well-being is still unknown. American studies report that, in the general population, the Internet is used to get additional information and/or advice about one’s own health [14], namely when facing a diagnosis and/or having to choose a treatment [15,16]. Sometimes the use of the Internet can postpone and even replace medical consultation and treatment [14]. Although teenagers do not usually make decisions about medical care autonomously, it is still relevant to assess the impact these online searches have on young adults’ health behaviors.

In France, several studies have focused on health information-seeking on the Internet [4,17-19]. However, to the best of our knowledge, no nationally representative sample has provided in-depth analysis of the behaviors and perceptions of young online health seekers by focusing on gender, income, and socioeconomic and health status.

### Objectives

The aims of this article are: (1) to provide information about the prevalence of Internet use for health-related purposes in France among young adults and define the sociodemographic, socioeconomic, and health-related profile of users, (2) to investigate the context and the impact of the information found on health-related behaviors, and (3) to assess the level of trust young adults have in the information found on the Internet.

## Methods

### Survey Methodology

Data were extracted from the National French Health Barometer survey conducted in 2010 by the French Institute for Prevention and Health Education (INPES) in consultation with the French Ministry of Health [20]. This survey was designed to measure the evolution of key indicators regarding health-related behaviors, attitudes, and opinions in the general population. Using a computer-assisted telephone interviewing (CATI) system, 27,653 people were interviewed from October 22, 2009 to July 3, 2010. Interviewers from a private survey firm were trained by the INPES to administrate this health-related survey.

We used a 2-stage random sampling design: (1) selection of households using random digit dialing covering all metropolitan French regions, and (2) random selection of one member of the household, using the method proposed by Kish [21]. Because of the increasing rate of households that have abandoned their landline telephones for cell phones, a cell-only sample was added (12% of the sample to keep the same rate as in 2010 in France). The cell-only sample was created independently from the landline sample by using the prefix (2 digits) assigned by the National Telecom Authority to each mobile phone provider. The remaining digits of the phone numbers (8 digits) were then randomly generated. If the respondent had a landline phone in his/her household, he/she was excluded from the cell-only sample. This development was essential to improve the coverage rate [22] because of the number of dwellings with a landline phone (87% in 2010 vs 96% in 1998). Thus, approximately 99% of the population was covered [4]. Details of the survey methodology have been published previously elsewhere [23,24].

If a household or individual refused to participate or could not be reached, they were not replaced in the study. Thus, specific efforts were made to successfully reach households and increase the response rate: a formal request to participate explaining the goals of the study was sent by mail before the first call (addresses were located from the landline phone numbers when available); unsuccessful calls were repeated after 30 and 90 minutes, on different days, and at different times to a maximum of 40 attempts for each generated phone number. Individuals who refused to participate were contacted a second time by specially trained interviewers. The overall refusal rate was 39%. All collected data were anonymous and self-reported. The mean duration of an interview was approximately 32 minutes for landline phones and 34 minutes for mobile phones.

This population-based survey procedure was approved by the French data protection authority (Commission Nationale de l’Informatique et des Libertés; CNIL), an independent administrative body that operates in accordance with the national data protection legislation, amended in 2004 specifically to protect citizens’ identities and privacy and ensure access to their own personal data.

From the initial nationally representative sample of 27,653 people aged 15-85 years, a random sample of 1052 young adults aged 15-30 years answered a set of specific questions on their use of the Internet as an information tool for health-related issues. In this paper, we will analyze this subsample (referred to as young adults or aged 15-30 years interchangeably).

Data were weighted by the number of telephone lines and eligible persons in the household. They were also adjusted to represent the French population structure (2008 census) according to age, gender, educational level, region of residence, and level of urbanization.

### Independent Variables

#### Sociodemographic Characteristics

Sociodemographic characteristics included the following: age group (15-19 years, 20-25 years, and 26-30 years), gender, socio-occupational status (categorized as manual workers, employees, intermediate occupations, executive and manager positions, and other), and income by consumption unit (adjusting for the household size and divided into quintiles). For those not working at the time of the interview, we used the head of household’s socio-occupational status.

#### Health Status

Respondents were questioned about their health status and if they had children or were expecting a child. The Duke Index, a validated tool containing 17 items that assesses general health status [25], was used to measure respondents’ health and well-being (score ranging from 0 to 100, later analyzed in tertiles). Psychological distress was measured using the 5-item Mental Health scale (MH-5; using a validated cut-off of 55), a specific section from the Short-Form 36 (SF-36) questionnaire, which is a validated, multipurpose, short-form health questionnaire with 36 questions [26]. Presence of a chronic disease was assessed by a self-reported answer (yes/no); if they answered yes, the disease had to be specified. Moreover, a variable named “fear of illness” was created as a score, analyzed in quartiles, summing answers to 10 questions concerning fear (not at all, a little, quite a few, a lot) of specific diseases or events (eg, traffic accidents, alcohol diseases, cancer, Alzheimer disease). Level of information on health issues was measured with a series of 13 questions (eg, Do you feel you are well informed about alcohol/tobacco/cancer...?). A 4-item scale (very well/well/bad/very badly informed) was used and the total score was analyzed using quartiles.

#### Trust in Internet Information

Survey respondents were asked about the credibility and trustworthiness of health-related information obtained on the Internet. Responses were categorized as reliable, somewhat reliable, not really reliable, not reliable at all, and “do not know.”

### Dependent Variables

#### Use of the Internet as a Source of Health Information

Survey respondents were asked whether they had ever used the Internet to search for information and advice about health and the frequency of their search(es) (eg, “During the past 12 months have you used the Internet to look for information or advice about health?” and “If so, how many times per week, month, or year?”). We also asked about the themes of their searches to a randomized subsample of 139 online health seekers. Answers to the latter question were grouped into 5 categories: general health and illnesses, medical news and treatments, mother and child health, health behaviors, and occasional diseases.

Individuals who never looked for health information on the Internet were asked if this was because they had enough information through other resources, they were not interested in getting health information, they were more confident in seeing a doctor for health-related questions, they were not confident with the information provided on the Internet, or they never thought about using the Internet to search for health-related information.

#### The Effect of Using the Internet on the Doctor-Patient Relationship

We subsequently asked the subsample of online health seekers if the information and advice found on the Internet had changed the way they take care of their health. In addition, they were asked if the use of the Internet led them to visit their doctor more often, less often, or as they did before using the Internet for health purposes. The context of the search was investigated by analyzing if respondents had often (compared with rarely or never) used the Internet for health purposes in the following situations: instead of seeing a doctor, before seeing a doctor, after having seen a doctor, and without link to any medical consultation.

### Statistical Analysis

Bivariate chi-square tests were performed on weighted proportions considering the following thresholds: .001, .01, and .05. Five multivariate logistic regression models were used to investigate whether risk factors (listed previously) were associated with (1) the use of the Internet for health purposes, and (2) the context and consequences of online health searches: Internet search instead of seeing a doctor, change in taking care of one’s own health, or having seen a physician less/more frequently. We estimated adjusted odds ratio (adjusted OR) and 95% confidence intervals (95% CI) based on the Wald test. The analyses were performed using R-3.0.1 software.

## Results

### Sample Characteristics

Data used in this analysis included 1052 individuals aged 15-30 years, of which 50.48% (531/1052) were men and 49.52% (521/1052) were women (Table 1). The mean age was 22.6 years (SD 0.18).

**Table 1:** Participant characteristics of landline and cell-only samples.

Characteristics		Total (N=1052)	Landline sample (n=733)	Cell-only sample (n=319)
**Gender, n (%)**				
	Men	531 (50.48)	369 (50.3)	162 (50.8)
	Women	521 (49.52)	364 (49.7)	157 (49.2)
**Age (years), n (%)**				
	15-19	322 (30.61)	289 (39.4)	33 (10.4)
	20-25	391 (37.17)	235 (32.1)	156 (48.9)
	26-30	339 (32.22)	209 (28.6)	130 (40.7)

### Use of the Internet as a Source of Health Information

#### Overview

**Figure 1:**
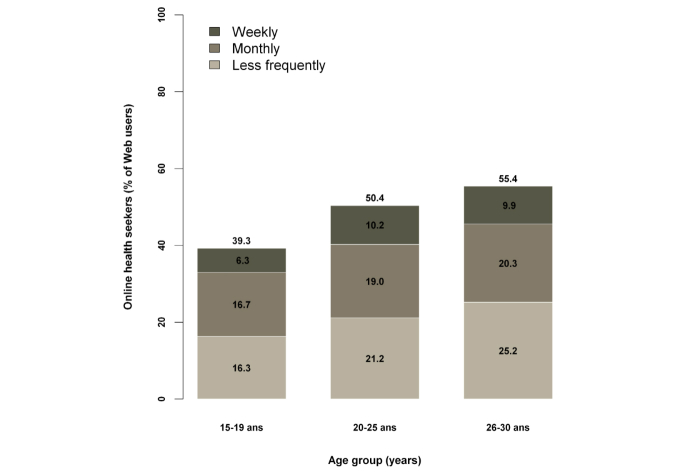
Web users and online health seekers by age group.

Almost all (977/1052, 92.87%) of our sample of young adults was comprised of Web users, and this proportion decreased slightly as age of respondents increased (from 96.2% to 90.1% for the 15-19 years and 26-30 years age groups, respectively) (see Figure 1).

#### Nonhealth Seekers

Among 977 Web users, 503 (51.5%) had never used the Internet to look for health information and advice during the past 12 months: 75.0% (377/503) explained being adequately informed by other sources, 74.1% (373/503) preferred seeing a doctor, and 67.2% (338/503) did not trust the information found on the Internet. Although there was no statistically significant difference by age, the youngest group (15-19 years) seemed more likely than others to be adequately informed through other sources and to have a distrust in information found on the Internet (Table 2).

**Table 2:** Reasons for not using the Internet for health information among Web users by age.

Reasons	Age group (years), n (%)				*P*
	15-30 (n=503)	15-19 (n=188)	20-25 (n=179)	26-30 (n=136)	
Adequately informed by other means and resources	377 (75.0)	148 (78.7)	128 (71.5)	101 (74.3)	.52
Not interested in this type of information	203 (40.4)	75 (39.9)	76 (42.5)	52 (38.2)	.80
More confident in seeing a doctor for this kind of information	373 (74.1)	138 (73.4)	131 (73.1)	104 (76.4)	.69
Distrust in the information provided by the Internet	338 (67.2)	135 (71.8)	114 (63.7)	89 (65.4)	.38
Do not know	244 (48.5)	102 (54.2)	78 (43.6)	64 (47.1)	.21

#### Health Seekers

Half of the Web users (474/977, 48.5%) used the Internet during the past 12 months to look for either information or advice on health: 8.9% (87/977) every week, 18.7% (183/977) every month, and 20.9% (204/977) less frequently (Figure 1). In summary, 45% of young adults used the Internet for health purposes.

Among Internet users, the use of the Internet for health purposes significantly increased with age: 39.3%, 50.4%, and 55.4% for the 15-19 years, 20-25 years, and 26-30 years age groups, respectively (*P*=.002). After adjusting for socioeconomic and health status, and all variables presented in Table 3, the logistic models showed that the likelihood of using the Internet for health purposes was higher among women compared to men (57.2% vs 39.7%; OR 1.8, 95% CI 1.3-2.3; *P*<.001; result not shown). Employees and manual workers were less likely than executives and managers to search for online health information and they actually surfed the Internet (whatever the reason) less frequently (among 15-30 year age group, 96.4% of executives and managers vs 92.6% of employees and 86.7% of manual workers). Finally, women with psychological distress (OR 2.0, 95% CI 1.0-4.0; *P=*.046), as well as pregnant women or women with at least 1 child (OR 1.8, 95% CI 1.1-2.7; *P=*.01) used the Internet for health purposes more frequently than others (Table 3).

**Table 3:** Factors associated with the use of the Internet for health purposes among Web users aged 15-30 years by gender.

Variables		Men (n=487)				Women (n=490)			
		n (%)	Adjusted OR	95% CI	*P*	n (%)	Adjusted OR	95% CI	*P*
**Socio-occupational status**					.04				.10
	Other	34 (54.0)	0.6	0.3, 1.4	.28	26 (47.6)	0.5	0.2, 1.4	.20
	Executives and managers	80 (54.5)	1			57 (64.8)	1		
	Intermediate occupations	129 (41.2)	0.7	0.4, 1.2	.15	163 (65.8)	0.9	0.4, 1.7	.64
	Employees	92 (33.6)	0.5	0.3, 0.9	.03	172 (51.4)	0.5	0.2, 0.9	.02
	Manual workers	152 (33.5)	0.4	0.2, 0.7	<.001	72 (54.7)	0.5	0.2, 1.0	.04
**Quality of life (Duke Index)**					.89				.03
	Third tertile (poor)	70 (40.6)	1			161 (62.3)	1		
	Second tertile (medium)	163 (37.9)	0.8	0.5, 1.5	.57	172 (61.3)	1.0	0.6, 1.6	.96
	First tertile (good)	254 (40.6)	1.0	0.6, 1.8	.93	157 (47.3)	0.6	0.4, 1.1	.09
**Chronic disease**					.23				.40
	Yes	40 (50.0)	1			46 (50.1)	1		
	No	447 (38.8)	0.7	0.3, 1.3	.22	444 (57.8)	1.3	0.7, 2.5	.38
**Psychological distress**					.51				.17
	No	467 (39.4)	1			426 (56.0)	1		
	Yes	20 (47.9)	1.6	0.6, 4.4	.34	63 (66.4)	2.0	1.0, 4.0	.046
**Having a child/being pregnant**					.12				.002
	No	432 (38.3)	1			356 (52.9)	1		
	Yes	55 (50.7)	1.7	0.9, 3.0	.09	134 (69.7)	1.8	1.1, 2.7	.01

^a^Logistic regression models were adjusted on all shown variables.

#### Themes of Online Health Searches

For the searched themes, those aged 15-30 years primarily looked for information on general health or specific diseases, especially flu or influenza (44.6%, 62/139). Themes searched by older people (31-85 years) concerned health behaviors, children’s health, and parental health. Women appeared to be particularly concerned with themes concerning children’s health and parental health (26.8%, 22/82) (Table 4).

**Table 4:** Health-related search themes according to gender among individuals aged 15-30 years.

Health topics	All, n (%) (n=139)^a^	Men, n (%) (n=57)	Women, n (%) (n=82)	*P*
General health and illnesses	62 (44.6)	27 (47.4)	35 (42.7)	.52
Children’s health and parental health	29 (20.9)	6 (10.5)	22 (26.8)	.06
Specific health problems	27 (19.4)	14 (24.6)	14 (17.1)	.31
Health behaviors	27 (19.4)	12 (21.1)	14 (17.1)	.59
Medical news/care	17 (12.2)	7 (12.3)	10 (12.2)	.88

^a^A randomized subsample of 139 online health seekers who were asked to specify the content of their searches.

#### Trust in Online Health Information

Approximately 80% (78.2%, 371/474) of online health seekers aged 15-30 years trusted the information they found on the Internet, even if 61.4% of them (291/474) qualified the information only as “somewhat” reliable, without significant differences according to age and gender. However, the opinion on the credibility of such information was associated with the way respondents took care of their own health. Among online health seekers, those who thought the information was not really reliable were less inclined to change the way they take care of their own health than those who found online information to be reliable (12.1% vs 39.2%, *P*<.001). Moreover people who found the information not really reliable did not decrease the frequency of their medical consultations (0.8% vs 8.1% for those who found the information reliable, *P*<.001).

#### The Effect of Online Health Searches on the Doctor-Patient Relationship

Table 5 illustrates the overall impact of Internet use on the young adults’ medical consultations. Almost 3 of 10 online health seekers aged 15-30 years reported having often used the Internet as a source of health information instead of seeing a doctor (29.9%, 142/474) or before seeing a doctor (28.7%, 136/474). By contrast, 16.7% (79/474) used the Internet after having seen a doctor, which significantly varied by age group: the 26-30 years group looked for information on the Internet after having seen a doctor significantly more often (22.4%, *P*=.03) than the 15-19 years (13.1%) and the 20-25 years (13.7%) groups.

Moreover, a total of 33.1% (157/474) of the 15-30 years group of health seekers stated they changed their way of taking care of their health. For 11.4% (54/474) of young online health seekers, the information found on the Internet in the past 12 months led them to see a doctor more often (4.9%, 23/474) or less often (6.5%, 31/474) than usual: the 20-25 years group tended to see their doctors less frequently (9.9%, 18/182) than the 15-19 years (4.1%, 5/122) and the 26-30 years (4.7%, 8/170) groups.

Finally, although 26.6% (126/474) looked for online health information without having had any kind of medical consultation, 33.1% (157/474) reported they modified the way they take care of their health based on the information they found on the Internet (no further significant difference by age group).

**Table 5:** Impact of online health searches among online health seekers by age group.

Impact of online health searches		Age group (years), n (%)				*P*
		15-30 (n=474)	15-19 (n=122)	20-25 (n=182)	26-30 (n=170)	
**Use the Internet for health purposes often” or very often...**						
	...instead of seeing a doctor	142 (29.9)	31 (25.4)	57 (31.3)	54 (31.8)	.42
	...before seeing a doctor	136 (28.7)	31 (25.4)	54 (29.7)	51 (30.0)	.69
	...after having seen a doctor	79 (16.7)	16 (13.1)	25 (13.7)	38 (22.4)	.03
	...not in relation to a medical consultation	126 (26.6)	28 (22.9)	48 (26.4)	50 (29.4)	.39
Use the Internet for health purposes has changed the way of taking care of one’s health		157 (33.1)	43 (35.2)	66 (36.3)	48 (28.2)	.35
**Use the Internet for health purposes has made medical consultations...**						
	...more frequent	23 (4.9)	7 (5.7)	10 (5.5)	6 (3.5)	.25
	...less frequent	31 (6.5)	5 (4.1)	18 (9.9)	8 (4.7)	
	...as often as usual	420 (88.6)	110 (90.2)	154 (84.6)	156 (91.8)	

Table 6 presents estimates of multivariate logistic regressions of 4 different outcomes assessing the perceived impact of online health searches. Young adults reporting the lowest level of economic resources were more likely to see their physician less frequently (OR 2.7, 95% CI 1.4-5.5; *P*=.004). Those reporting poor quality of life according to the Duke scale (third tertile) were more likely to search often for health information on the Internet instead of seeing a doctor than those reporting good quality of life (OR 2.3, 95% CI 1.4-3.7; *P*<.001). They were also more likely to change the way they take care of their own health (OR 1.8, 95% CI 1.1-2.9; *P*=.009) and were more likely to see their physician more frequently (OR 2.7, 95% CI 1.0-7.4; *P*=.048). Finally, people with psychological distress, fearing illnesses (OR 3.3, 95% CI 1.0-10.5; *P*=.04), and those less informed about diseases (OR 3.2, 95% CI 1.1-9.0; *P*=.02) tended to increase the frequency of their consultations because of their online health searches.

**Table 6:** Factors associated with the context and consequences of online health searches, with odds ratios adjusted (adj OR) on all shown variables (N=474).

Factor		n	Internet search instead of seeing a doctor			Change in taking care of one’s own health			Has seen the physician less frequently			Has seen the physician more frequently		
			Adj OR	95% CI	*P*	Adj OR	95% CI	*P*	Adj OR	95% CI	*P*	Adj OR	95% CI	*P*
**Gender**														
	Male	193	1			1			1			1		
	Female	281	0.7	0.5-1.1	.11	0.6	0.4-0.8	.003	0.5	0.3-1.1	.06	1.9	0.8-4.7	.15
**Low income (1st quintile)**														
	No	363	1			1			1			1		
	Yes	111	1.0	0.71.6	.89	1.4	0.9-2.1	.21	2.7	1.4-5.5	.004	1.7	0.8-3.7	.23
**Quality of life (Duke Index)**														
	Third tertile(poor)	130	2.3	1.4-3.7	<.001	1.8	1.1-2.9	.009	1.2	0.5-2.7	.69	2.7	1.0-7.4	.048
	Second tertile (medium)	162	1.3	0.8-2.0	.23	1.1	0.7-1.7	.79	0.7	0.3-1.7	.48	1.9	0.7-5.3	.24
	First tertile (good)	182	1			1			1			1		
**Chronic disease**														
	Yes	44	1			1			1			1		
	No	430	0.8	0.4-1.4	.41	0.7	0.4-1.2	.14	0.9	0.3-2.6	.81	0.7	0.2-2.0	.42
**Psychological distress**														
	No psychological distress	419	1			1			1			1		
	Psychological distress	55	2.2	1.3-3.7	.004	1.0	0.6-1.8	.77	1.1	0.4-3.1	.73	3.2	1.4-7.4	.003
**Fear of illness**														
	First quartile (less afraid)	115	1			1			1			1		
	Second quartile	129	1.4	0.8-2.3	.22	1.0	0.6-1.7	.94	1.3	0.5-3.0	.53	1.4	0.4-5.0	.64
	Third quartile	129	1.3	0.8-2.1	.39	1.6	1.0-2.7	.04	0.5	0.2-1.4	.19	1.9	0.6-6.6	.26
	Fourth quartile (more afraid)	102	1.2	0.7-2.0	.64	1.7	1.0-2.9	.06	0.9	0.3-2.3	.65	3.3	1.0-10.5	.047
**Level of information on health issues**														
	First quartile (well informed)	96	1			1			1			1		
	Second quartile	129	1.2	0.7-2.1	.60	1.1	0.6-1.8	.80	1.4	0.4-4.3	.56	0.8	0.2-2.9	.75
	Third quartile	122	1.6	0.9-2.8	.12	0.6	0.3-1.0	.048	1.2	0.4-3.9	.88	0.7	0.2-2.6	.65
	Fourth quartile (poorly informed)	127	1.9	1.1-3.3	.03	1.0	0.6-1.7	.96	2.4	0.8-6.8	.16	3.2	1.1-9.0	.02

## Discussion

### Characteristics of Online Health Seekers

According to the national Health Barometer 2010 survey data based on a random and representative sample of the French population, 45% of young adults aged 15-30 years used the Internet in the past 12 months to seek health information. This result is in-line with a study from the French National Institution of Statistics and Economical Studies (INSEE) in 2010 examining the same question, among others, but over a period of 3 months [17]. At the international level, this proportion is lower than in other countries. A survey performed in 7 European countries (Norway, Denmark, Germany, Greece, Poland, Portugal, and Latvia) in 2005 found that, on average, 63% of individuals aged 18-29 years were online health seekers [27]. The replication of this study in 2007 showed that this behavior was growing in all age groups [28]. Another survey carried out in 2010 in Italy showed that 60% of young Italian males and 65% of young Italian females (between 18-29 years) used the Internet for health-related purposes [29]. In the United States, the Pew Research Center’s Internet & American Life Project showed that in 2012, 72% of people aged 18-29 years were online health seekers [30]. Considering the 18-29 years group in our own study, the proportion of online health seekers only increased to 48%. However, this apparent lower proportion of young online health seekers in France may be because of an underestimation in our study as a result of formulation issues (see Limitations). Moreover, the aforementioned international surveys exclusively focused on online health information seeking and do not aim at being representative of the general population, which is the case with the Health Barometer 2010.

Online health seekers aged 15-30 years were more likely to be women than men and have a position as an executive or manager rather than being employees or manual workers. The literature confirms that gender, occupation, and socioeconomic status are the main factors discriminating the Internet use for health concerns in the general population [22-26]. Those factors have also been found in very different contexts and countries (eg, Saudi Arabia, Brazil, or Japan) [31-33].

Regarding the gender effect, this result is not specific to the Internet use for seeking answers to health questions because women, in general, tend to be more interested in health than men [34]. However, it is worth noting that women’s use of the Internet for health advice or information seeking has empowered them and changed their relationship with health care providers [35]. Moreover, other results showed that women were more likely than men to seek help for someone else [36].

The association with socioeconomic status is also consistent with the fact that the Internet, as any technological innovation, tends to primarily benefit the wealthier and/or more educated [37-39], and in this case reinforce the inverse information law (as an extension of the inverse care law as defined in 1971 [40]): the availability and use of health information (and the ability to use it properly) tends to vary inversely with the need of the population. Understanding the sociodemographic and socioeconomic profiles of online health seekers could, therefore, help improve the quality of online information and tools (eg, by adjusting the level of literacy required) and produce age- or gender-specific online supports.

Although our sample is relatively homogeneous regarding age, we saw that the proportion of Web users slightly decreased with increasing age, whereas the proportion of online health seekers increased. This was previously found in the general population [3,41-44] and could probably be related to the ambiguity of the age effect [45]. On the one hand, younger people, namely adolescents, have more access to the Internet and have better and more Web-related skills [46-48]. On the other hand, they are less concerned with health problems, the latter increasing with age and impacting those generations who are a priori less at ease with using the Internet [49,50]. Our results suggest that national and regional health agencies could develop health promotion campaigns and programs targeting young adults to bridge the gap between their low level of knowledge regarding health issues and the increasing prevalence of lifestyle diseases [51].

Some health-related factors were also associated with the use of the Internet to search for health-related information. Our data did not show significant associations with general health status (measured in our study through chronic disease or quality of life) as often shown in the general population [27,45,52,53]. However, 2 specific conditions were found to be associated with online health searches. Psychological distress appeared related to searching for health information on the Internet. This could be explained by the fact that a specific condition, rather than a perceived general health status, increases the interest and the need to search for specific information or treatment. Moreover, anxiety itself could lead these people to look for further health information or to verify information after a medical consultation. Furthermore, the confidentiality of the Internet could represent an advantage for its use as a tool to obtain information on stigmatizing issues, such as many mental health illnesses. This could explain, at least partially, why the use of the Internet for health purposes is positively associated to poor mental health but not to physical health.

The last factor associated with the use of the Internet for health-information seeking is having or expecting a child, especially among women. Again, this might be related to the fact that women, more than men, still tend to take care of the family’s health. The interest in health information dealing with parenthood is clear when we look at the most frequent themes. Questions about mother’s and child’s health are indeed the most frequently mentioned topics among the young Web users (21%) after general health and illnesses (45%). These findings are consistent with those in other countries in Europe, such as Italy [54]. This noticeable interest in parenthood probably represents an interesting starting point for health promotion providers and policy makers in France. The creation of specific websites on this topic could meet the needs of parents and provide them with validated information.

### Context and Impact of Online Health Searches

With regard to the context of health-information seeking on the Internet, three-quarters of online health seekers reported having made their Internet searches in conjunction with a medical consultation, either before (eg, to see if a consultation is needed or to get prepared to an eventual treatment) or after (eg, to get additional information or seek for alternative treatments). More interestingly, approximately 3 in 10 young adults reported having looked for health information on the Internet instead of seeing a doctor. This behavior could fit with a search cost model using the Internet as a resource to reduce health care and information search costs [55]. By finding reassuring information about specific and precise questions on the Internet, young adults could save the money and time of any medical consultation.

Moreover, one-third of the 15-30 years group of online health seekers reported having modified the way they take care of their health after their Internet searches. It is possible that the changes in question reflect an increased distancing from health professionals, which may lead young adults to follow advice against public health rules (eg, purchasing medicines on the Internet or trusting uncontrolled therapies). Conversely, these findings could be positive if people have been trained and influenced by trustworthy online information campaigns or prevention programs. In both cases, these results confirm the idea that the Internet potentially supports the dissemination of health information with an impact on young adults’ health, as well as the importance of promoting labels to guarantee the reliability of the information provided on commercial websites, or to train users to read in a critical manner.

### Trust in Online Health Information

Although two-thirds of young people did not look for health information on the Internet because of their distrust in this kind of information, the majority of young online health seekers (approximately 80%) trusted the information they found on the Internet. This could be worrisome because the quality and validity of health information on the Internet varies a lot in France, as it does in other countries [8-12]. For instance, a 2009 study conducted in the United Kingdom showed that only 4 of 10 websites provided correct information regarding pediatric issues [11]. However, our statistical analyses showed that the opinion on the credibility of online information is linked to the repercussions Internet searches have on the way people take care of their own health. In fact, those who trusted the less health-related information are also those who stated less change in their health and medical behavior because of their Internet searches. In any case, it is important to underline that we do not know to what extent the level of trust in the information found online is related to the actual validity of the information.

Finally, if young adults feel comfortable using the Internet, they may have difficulty judging the quality of health-related information or they may not be aware of quality labels. Therefore, it is fundamental to help young people to find and use the most valid online health information. Several strategies can be developed to reach this goal. On the one hand, institutional websites need to be created—or the promotion of labels on other websites—where health information is clearly thought through, well planned, referenced, and safely managed [56]. This process is already in place in the United States and Australia [2,3,43], where young Web users represent a large proportion of online health seekers. In France, the INPES also developed many validated information resources on the Internet and social networks dedicated to young people during the last 10 years. This agency also promoted its reliable online resources through other media (eg, television, schools). In a complementary manner, it seems useful to offer health-related educational programs and e-learning activities to young adults.

Another strategy to ensure young adults get valid information about health issues is to target their main way of using the Internet, namely social networks. There is a growing use of social networks for health promotion purposes and the literature shows that those interventions are effective in some fields, such as sexual health promotion [57-59]. That is also something the INPES has tried to develop in recent years. Social networks could also be established as a place for physicians and health professionals to help their patients wade through online information and make recommendations on reliable sources. It is then necessary to develop the monitoring, validation, and labeling of new tools created by health professionals and experts. Professional organizations could attempt to build digital resources for young people and work with them in a collaborative manner, as the nature of Web 2.0 suggests.

### Limitations

Analyses were based on a large sample representative of the French population. The methodology of the survey has been validated and interviews were conducted by trained interviewers. However, several limitations deserve attention in the interpretation of our findings. The response rate was 61%, which is satisfactory compared with other health surveys in France, but lower than the rates obtained in other epidemiologic surveys, such as the National Epidemiologic Survey on Alcohol and Related Conditions (NESARC) [60]. However, selection bias cannot be ruled out and some populations, especially the most deprived ones (ie, homeless people), are likely to be underrepresented, although some were interviewed as a result of the sample based on mobile phone numbers.

Among the limits of our approach, it is also necessary to underline that our data do not allow for the distinction among Web sites, blogs, and social networks. People who use these tools do not necessarily all have the same approach; therefore, they might not have the same profile (eg, socioeconomic status and health behaviors). It is possible that a part of the young adults who looked for information about health behavior answered no to the question “have you used the Internet to look for health information or advice.” Tobacco smoking, sexual behaviors, drug consumption, or sleep habits may indeed not be perceived as health behaviors by young people who might not perceive the health consequences of their behaviors, particularly those that will occur in the long term.

### Conclusions

Our study shows that in France in 2010, almost all individuals aged 15-30 years were Web users, and approximately half of them used the Internet to look for health information for themselves, their relatives, or nobody in particular. These results justify the increasing effort over the past several years by health promotion stakeholders in designing specific e-tools, such as the development by agencies or labeled stakeholders of websites or Facebook pages dedicated to adolescents and young adults, of online publishing of video events (eg, INPES manga [61] aimed at preventing the initiation of smoking), or the development of smartphone apps (eg, Alcoholometer app to estimate daily alcohol consumption).

To conclude, the Internet is assuming an increasingly important role in its young users’ lives and is increasingly becoming one of the major health information mediums in many countries. This explains why effective health interventions for young people should not avoid online tools. Given the results of this study, France is expected to maintain enhancing the number and quality of health-related websites especially addressed to individuals aged 15-30 years. It is incumbent to find more creative ways to inform young people about health and health care in ways that reflect their own style and culture.

## Abbreviations

CATI: computer-assisted telephone interview
CNIL: Commission Nationale de l’Informatique et des Libertés
HAS: Haute Autorité de Santé
HON: Health on the Net
INPES: Institut National de Prévention et d’Education pour la Santé
INSEE: Institut National de la Statistique et des Etudes Economiques
MH5: 5-item Mental Health scale from the SF-36
SF-36: 36-item Short-Form health questionnaire
